# Psychological Inflexibility as a Predictor of Sexual Functioning Among Women with Vulvovaginal Pain: A Prospective Investigation

**DOI:** 10.1093/pm/pnaa042

**Published:** 2020-03-18

**Authors:** Pernilla Maathz, Ida K Flink, Linnea Engman, Johanna Ekdahl

**Affiliations:** 1 Department of Psychology, Uppsala University, Uppsala, Sweden; 2 Center for Health and Medical Psychology (CHAMP), School of Law, Psychology and Social Work, Örebro University, Örebro, Sweden; 3 Department of Psychology, Mid Sweden University, Östersund, Sweden

**Keywords:** Vulvovaginal Pain, Psychological Inflexibility, Sexual Functioning, Genital Pain

## Abstract

**Objective:**

Persistent vulvovaginal pain affects many women and often has adverse effects on sexual functioning. Psychological inflexibility related to pain is associated with distress and functional disability across different types of chronic pain conditions, but little is known about the role of psychological inflexibility in vulvovaginal pain. The present study examines psychological inflexibility related to pain as a predictor of sexual functioning over time among women with vulvovaginal pain.

**Methods:**

Questionnaires including measures of psychological inflexibility, pain severity, and sexual functioning were administered to female university students at two points in time. One hundred thirty women with vulvovaginal pain responded to the questionnaire at baseline and at follow-up after 10 months. A multiple regression model was used to explore psychological inflexibility and pain severity as predictors of sexual functioning at follow-up.

**Results:**

Higher levels of psychological inflexibility and more severe pain at baseline were associated with poorer sexual functioning 10 months later. In analysis adjusting for baseline levels of sexual functioning, psychological inflexibility was the only significant predictor of sexual functioning at follow-up.

**Conclusions:**

The findings provide preliminary evidence that psychological inflexibility is associated with sexual adjustment over time among women with vulvovaginal pain and point to the relevance of further examinations of the psychological inflexibility model in the context of vulvovaginal pain.

## Introduction

Persistent vulvovaginal pain is a common type of pain, affecting ∼8% of adult women [1]. Like other pain conditions, vulvovaginal pain is associated with elevated levels of psychological distress [2]. As the pain is triggered by sexual activities that involve pressure to the vulva, particularly intercourse, a large number of women also report that it has significant adverse effects on their sex life [3]. Having vulvovaginal pain is associated with lower sexual satisfaction [4] and functioning, including problems with sexual desire, arousal, lubrication, and orgasm [5]. In the greater literature on chronic pain, there is substantial evidence that psychological processes play an important role in the development of pain and in long-term adjustment to pain [6,7]. Although psychological responses known to predict prolonged pain and dysfunction related to chronic pain have been shown to also influence vulvovaginal pain and its impact on sexual functioning [8–10], they are still sparsely examined in this type of pain.

In the wider field of chronic pain, psychological inflexibility is increasingly acknowledged as a predictor of pain-related dysfunction and distress. It is a core concept in Acceptance and Commitment Therapy (ACT) [11,12], which is considered a well-established treatment for chronic pain (APA, division 12). Psychological inflexibility refers to persistent patterns of actions that are guided predominantly by inner experiences, typically with the aim to avoid certain thoughts, feelings, and sensations, in a way that is disconnected from direct environmental contingencies and personal goals and values [13]. In chronic pain, psychological inflexibility entails behaviors that bring short-term relief, such as physical inactivity and avoidance of activities that are associated with experiencing pain, but also rumination and attempts to distract from or suppress pain. Although these behaviors aim to reduce distressing inner experiences (i.e., experiential avoidance), they often have the counterintuitive effect of increasing their salience, and suffering is maintained or exacerbated over time as these behaviors hinder meaningful life activities. Psychological flexibility, on the other hand, is characterized by acceptance of inner experiences, by awareness of the present moment and the opportunities it holds, and by engagement in actions that are consistent with personal goals and values [12].

Consistent with this theoretical framework, prior studies have found that psychological inflexibility predicts quality of life and functional disability and distress among individuals with chronic pain [14,15]. Further, it has been reported repeatedly that greater acceptance of pain is associated with less emotional distress and disability [16–18]. Reductions in psychological inflexibility have also been found to mediate changes in pain-related functioning, distress, and quality of life following treatment [19,20]. When it comes to vulvovaginal pain, psychological inflexibility may lead to a general avoidance of sexual activity as it is associated with experiencing pain, thereby interfering with valued aspects of intimate relationships and affecting sexual satisfaction. Also, a focus on controlling or distracting from pain during sexual activity may have the effect of intensifying pain [21], as well as decreasing the ability to connect with and respond to direct contingencies such as present moment experiences of pleasure, resulting in more negative experiences of sex and declining sexual functioning. Accordingly, recent studies show that avoidance of sexual relations is associated with reduced sexual functioning over time among women with vulvovaginal pain [22] and that acceptance of pain is associated with lower pain intensity and greater sexual functioning and satisfaction [23].

Considering that psychological inflexibility has emerged as a relevant predictor of adjustment to chronic pain and that previous studies link both acceptance and avoidant behavior to sexual adjustment among women with vulvovaginal pain, the role of psychological inflexibility should be examined further in this context. Moreover, there is a need for longitudinal studies to advance the understanding of the relationship between vulvovaginal pain and sexual functioning and to clarify the association between pain severity and psychological inflexibility. Hence, the aim of this prospective study was to investigate psychological inflexibility and pain severity as predictors of sexual functioning over time among women with vulvovaginal pain.

## Methods

### Design

The current study is based on data collected within the Sex and Pain (SAP) project. The SAP project is a longitudinal investigation examining potential predictors of development of vulvovaginal pain among women in general and predictors of sexual functioning over time among women with vulvovaginal pain. Data were collected at three points in time over a period of 10 months using questionnaires. This study uses data only from women with vulvovaginal pain, collected at the first (baseline) and third (follow-up) measurement points.

### Procedure

Participants were recruited in a classroom setting at two universities in Sweden. In total, 66 classes were approached, and the women in the classes were invited to stay and receive information about the study. Those who agreed to participate in the study handed in signed informed consent forms and received a copy of the questionnaire. They were given the choice of completing the questionnaire in the classroom or bringing it with them to complete at their own convenience and returning it in a closed box at the research institute. At the following measurement points, the informed consent forms and questionnaires were mailed to the participants for them to fill out and return in prepaid envelopes. Nonresponders were sent reminders by e-mail after two and four weeks. Women who participated in the first measurement point received coffee coupons valid in the university cafeterias as compensation, and those who responded at the last measurement point were compensated with a cinema ticket. The study was approved by the Regional Ethical Review Board in Uppsala, Sweden (D Number 2014–407).

### Participants

To participate in the study, female gender and age 18–35 were required. Following the recommendations by Green [24] for determining sample size for regression analysis, it was estimated that 107 participants with vulvovaginal pain were required to examine the study variables in the SAP project. To achieve this, given a prevalence of vulvovaginal pain of 15–20% of the student population, it was estimated that a minimum of 535 participants would be needed. Taking into account the dropout rates in longitudinal survey studies, the aim was to include twice this number of participants.

In total, 1,021 women consented to participate and completed the questionnaire at the first measurement point, and 546 (53.5%) responded at 10-month follow-up. There were no significant differences between the group of participants who responded at the first measurement point only and the group who also responded at follow-up on any of the variables included in the analysis: age (*t*(1,017) = 1.33, *P* = 0.18), psychological inflexibility (*t*(999) = 0.80, *P* = 0.42), sexual functioning (*t*(989) = 1.01, *P* = 0.31), or the occurrence of vulvovaginal pain (χ^2^(1, N* *=* *1,010) = 0.70, *P* = .41). Among the participants who responded at the first measurement point, 230 women (23.8%) met the criteria used to assess vulvovaginal pain, detailed below. At follow-up, 130 of the women who met criteria for vulvovaginal pain responded to the questionnaire, and this group constituted the sample used in this study. Sample characteristics at baseline are displayed in Table 1.

**Table 1:** Participant characteristics at baseline (N = 130)

Age, y		
Mean (SD)	22.70	(2.74)
Children		
Yes, No. (%)	9	(7)
No, No. (%)	119	(93)
Partner		
Yes, No. (%)	93	(72)
No, No. (%)	33	(26)
Other, No. (%)	3	(2)
Gender of partner		
Female, No. (%)	5	(5)
Male, No. (%)	90	(94)
Other, No. (%)	1	(1.0)

### Measures

#### Vulvovaginal Pain

Participants who met the following criteria were categorized as women with vulvovaginal pain and were included in the study: 1) reported that they had been troubled by persistent pain to the vulvar area during intercourse or upon touch for the past three months or longer; the question used to assess this criterion was phrased based on findings from a study investigating the correspondence between self-reported vulvar pain and clinically confirmed vestibulodynia [25]; 2) reported that pain or discomfort had occurred repeatedly over the last four weeks. This criterion corresponds to a score <5 on the first question of the Female Sexual Function Index (FSFI) Pain subscale [26].

#### Sexual Function

The Female Sexual Function Index (FSFI) [26] was used to assess sexual function. The original scale contains 19 questions on six subscales: Desire, Arousal, Lubrication, Orgasm, Sexual Satisfaction, and Pain (e.g., “Over the past four weeks, how often did you feel sexual desire or interest?”). Responses are scored from 0 *or* 1 to 5, depending on item, with higher scores representing greater sexual functioning and a score of 0 indicating no sexual activity during the last month. In this study, sexual function was assessed using the total score on the FSFI with the Pain subscale subtracted, giving a score ranging between 2 and 30 points. The FSFI has shown good psychometric quality [26,27], also when evaluated in a Swedish sample [28]. Internal consistency was α = 0.94 at baseline and α = 0.95 at follow-up in this study.

#### Pain Severity

The Pain subscale of the FSFI [26] was used to assess pain severity in this study. The subscale consists of three items relating to the frequency of pain and pain intensity (e.g., “Over the past four weeks, how often did you experience pain or discomfort during vaginal intercourse?”). Answers are given on a scale ranging from 0 to 5, with 0 indicating no attempts at vaginal intercourse. The total score ranges from 0 to 6. To facilitate interpretation of the results from this study, scores were reversed so that higher scores reflected more severe pain. The internal consistency of the Pain subscale was α = 0.92 at baseline and α = 0.95 at follow-up in this study.

#### Psychological Inflexibility

To assess psychological inflexibility, the Psychological Inflexibility in Pain Scale (PIPS) [14,29] was used. The instrument consists of 12 items on two subscales: Avoidance of Pain and Cognitive Fusion with Pain (e.g., “I do not do things that are important to me to avoid feeling my pain”). Items are rated on a seven-point scale, giving a total score of 12–84, with higher scores representing greater psychological inflexibility. The instrument has been evaluated in different chronic pain samples and has shown satisfactory psychometric quality [14,29,30]. Internal consistency was α = 0.91 at baseline and follow-up in this study.

### Statistical Analyses

Before analysis, missing values were replaced with the individual’s mean score of the available subscale items, allowing one missing item on subscales with four or more items. The missing data did not exceed 2% on any of the variables. Descriptive statistics were calculated to summarize data, and a series of Pearson product–moment correlations were performed to examine concurrent and longitudinal relationships between the variables in the study.

To investigate the relationship between pain-related psychological inflexibility, pain severity, and sexual functioning over time, hierarchical linear regression analysis was used. In a model predicting sexual functioning at follow-up, sexual functioning at baseline was entered in the first step, followed by pain severity and psychological flexibility at baseline in the second and third steps, respectively. In all analyses where the FSFI was included, participants were excluded who reported no sexual activity at baseline (N* *=* *8) to reduce the risk of confounding sexual inactivity with sexual dysfunction. All analyses were performed using the IBM Statistical Package of Social Sciences (SPSS) 24.0.

## Results

### Concurrent and Longitudinal Correlations Between Psychological Inflexibility, Pain Severity, and Sexual Function

The relationships between psychological inflexibility, pain severity, and sexual functioning among women with vulvovaginal pain were initially investigated by analyzing the correlations between the variables at baseline. Results from the analysis and descriptive statistics are displayed in Table 2. There was a positive correlation between psychological inflexibility and pain severity at baseline (*r* = 0.27, *P* = 0.003), indicating that women with greater psychological inflexibility experience more severe pain. Women with more severe pain experienced poorer sexual functioning (*r* = –0.41, *P* < 0.001), but there was no significant association between psychological inflexibility and sexual functioning at baseline.

**Table 2:** Descriptive statistics and correlations between variables at baseline

	M (SD)	Sexual Functioning	Pain Severity
Sexual functioning^†^	20.72 (6.26)		
Pain severity^‡^	2.54 (1.69)	–0.44***	
Psychological inflexibility^§^	37.21 (14.06)	–0.03	0.25**

Significance levels relate to two-tailed Pearson product–moment correlations.

* *P* < 0.05;

** *P* < 0.01;

*** *P* < 0.001.

† Female Sexual Function Index.

‡ Female Sexual Function Index Pain subscale.

§ Psychological Inflexibility in Pain Scale.

To examine the associations between psychological inflexibility, pain severity, and sexual function over time, correlations between the variables at baseline and follow-up were investigated. Longitudinal correlations are presented in Table 3. There were significant negative correlations between psychological inflexibility and sexual functioning at follow-up *r*= 0.20, *P* = 0.02), and between pain severity and sexual functioning (*r*= 0.24, *P* = 0.008), with women with higher levels of pain and psychological inflexibility experiencing poorer sexual functioning 10 months later. Further, psychological inflexibility, sexual functioning, and pain severity at baseline were significantly correlated with pain severity at follow-up. Women with poorer sexual functioning and higher levels of psychological inflexibility and pain experienced more severe pain later. However, pain severity at baseline was not associated with psychological inflexibility at follow-up.

**Table 3:** Correlations between sexual functioning, pain severity, and psychological inflexibility at baseline and follow-up

	Follow-up		
Baseline	Sexual Functioning^†^	Pain Severity^‡^	Psychological Inflexibility^§^
Sexual functioning^†^	0.58***	–0.37***	–0.01
Pain severity^‡^	–0.24**	0.48***	0.15
Psychological inflexibility^§^	–0.20*	0.22*	0.56***

Significance levels relate to two-tailed Pearson product–moment correlations.

* *P* < 0.05;

** *P* < 0.01;

*** *P* < 0.001.

† Female Sexual Function Index.

‡ Female Sexual Function Index Pain subscale score.

§ Psychological Inflexibility in Pain Scale score.

### Psychological Inflexibility and Pain Severity as Predictors of Sexual Functioning over Time

The results from the hierarchical multiple regression analysis investigating psychological inflexibility and pain severity as predictors of sexual functioning over time showed that baseline sexual functioning and psychological inflexibility predicted sexual functioning at follow-up (*β* = 0.58, *t* = 3.4, *P* < 0.001, and *β* = –0.18, *t* = –2.31, *P* = 0.023, respectively). Pain severity at baseline did not uniquely predict sexual functioning at follow-up. The full model accounted for 36% of the variance in sexual functioning at follow-up (*R*^2^ = 0.36, *F*(3, 113) = 21.33, *P* < 0.001). The results are presented in Table 4.

**Table 4:** Hierarchical multiple regression analysis examining sexual functioning, pain severity, and psychological inflexibility at baseline as predictors of sexual functioning at follow-up

Model	Adj. *R*^2^	*b*	SE	*β*	*P*
Step 1	0.33				
Sexual function*		0.67	0.09	0.58	<0.001
Step 2	0.32				
Sexual function*		0.67	0.10	0.58	<0.001
Pain severity^†^		0.04	0.31	0.01	0.902
Step 3	0.35				
Sexual function*		0.69	0.10	0.59	<0.001
Pain severity^†^		0.26	0.32	0.07	0.421
Psychological inflexibility^‡^		–0.08	0.03	–0.18	0.023

In this model, sexual functioning was entered in the first step, followed by pain severity and psychological inflexibility in the second and third steps, respectively.

* Female Sexual Function Index baseline score.

† Female Sexual Function Index Pain subscale baseline score.

‡ Psychological Inflexibility in Pain Scale baseline score.

## Discussion

In this study, we used a prospective design to examine the association between pain-related psychological inflexibility, pain severity, and sexual functioning over time among women with vulvovaginal pain. We found no concurrent association between psychological inflexibility and sexual functioning at baseline, but women with higher levels of psychological inflexibility at baseline reported poorer sexual functioning later. Pain severity was associated with sexual functioning both concurrently and longitudinally. In analysis adjusted for baseline sexual functioning, psychological inflexibility predicted sexual functioning 10 months later. Pain severity did not emerge as an independent predictor of sexual functioning over time. The results indicate that pain-related psychological inflexibility is linked to the sexual difficulties women with vulvovaginal pain develop, with women with higher levels of psychological inflexibility experiencing poorer sexual functioning over time. Although high levels of psychological inflexibility at baseline were associated with more severe pain at follow-up, pain severity at baseline was not significantly related to psychological inflexibility at follow-up. This finding suggests that psychological inflexibility is not a reaction to more severe pain but a precursor of elevated pain.

Psychological inflexibility is gaining recognition as a predictor of adjustment to chronic pain, and the results from this study indicate that the same process also predicts sexual adjustment among women experiencing vulvovaginal pain. Moreover, the results are consistent with another regularly reported finding in chronic pain research: When psychological inflexibility is taken into consideration, pain intensity does not seem to be a key factor in explaining pain-related functioning or well-being [31,32]. Also in the context of vulvovaginal pain, there are several studies reporting that the value of pain intensity as a predictor of sexual function and satisfaction is limited [33–35]. The findings indicate that the level of sexual dysfunction of women with vulvovaginal pain is not determined simply by the level of pain that they experience but more so by their way of responding to that experience, and that this process may be similar in persistent vulvovaginal pain and other chronic pain conditions.

When it comes to ways of responding to pain, previous research has linked both pervasive avoidance of sexual activity and endurance in painful sexual activities to low sexual well-being [9,36]. Although avoidance and endurance may appear to be distinct or even opposite behavioral responses, a recent study demonstrates that some women report high levels of both avoiding and enduring painful sex and that they have the most adverse outcomes [22]. Even if these behaviors take different forms, they may be similar in function as they both in a given context can serve to avoid an unwanted outcome. For example, women who endure painful sex mention that they do it to please their partners [37] and to avoid shame, guilt, or fear of losing a partner [38]. Although avoidance motivation for sexual activity has been linked to poor outcomes, women who are motivated more by intimacy, pleasure, or other positive outcomes have been found to experience higher relationship and sexual satisfaction [39,40]. These results are well in line with the functional perspective provided by the psychological inflexibility model, suggesting that psychosexual functioning does not depend on any particular behavior in itself, but on whether that particular behavioral pattern is consistent or in conflict with personal values.

The findings from this study indicate that women with vulvovaginal pain may be helped to better sexual functioning by interventions based on ACT. ACT has been shown effective when it comes to other chronic pain conditions [41], and previous research has suggested that acceptance of the inner experience, present moment contact, and affirmation of values may be beneficial also to women with vulvovaginal pain [23,42,43]. In the context of vulvovaginal pain, acceptance may allow individuals to engage in valued activities in circumstances where interpersonal fears or pain may show up. For example, adding other sexual activities than intercourse to the sexual repertoire may come with feelings of insecurity, and feelings of guilt or shame may be associated with sometimes not living up to a partner’s expectations, but doing and feeling those things may also be part of a sex life that reflects personal values and gives pleasure. Given that, it is important to note that acceptance is not the same as having intercourse despite pain. Contact with the present moment can increase the awareness of pleasurable body sensations [44] and sexual stimuli present in the moment, which may enhance feelings of arousal and give a more positive experience of sexual activity [45]. If women are able to track their moment-to-moment experiences and adjust their sexual practices according to what gives pleasure, it may result in a broader range of sexual activities than merely intercourse and may increase sexual satisfaction.

Some methodological limitations to the current study should be noted. This study used a student sample, which may limit the generalizability of the results, particularly to other age groups. To examine if the findings translate to women with vulvar pain syndromes, studies in clinical samples will be needed. Further, there is a lack of information about response rate and the characteristics of the women who chose not to participate in the study. Other limitations relate to the measures used in this study. First, the instrument used to measure psychological inflexibility, the PIPS, was developed for use with people with chronic pain in general and has not been psychometrically evaluated in a specific population with vulvovaginal pain. Accordingly, the mean scores on the PIPS are considerably lower in the sample in this study than in previous chronic pain samples [14,46], while a previous study using an adapted measure of pain-related avoidance show levels comparable to those found in other chronic pain samples [10]. We cannot rule out that a scale adapted for this specific population would be more sensitive to the relationship between psychological inflexibility and sexual dysfunction. Second, the instrument used to assess sexual function, the FSFI, is a very well-established measure of female sexual function, but the procedure used to score the FSFI has received some criticism [47], with implications for the interpretation of results from this study. Some of the items of the FSFI have a response option for “no sexual activity during the last four weeks,” and that response option gives a score of 0. Thus, participants reporting no sexual activity will have low scores, and lower scores are interpreted as poorer sexual functioning. This, however, causes concerns that poor sexual functioning may be confounded by sexual inactivity that occurs for other reasons, and therefore participants in this study who reported no sexual activity at baseline were excluded from analysis. That is a conservative way of addressing the issue and comes with the disadvantage of, potentially, excluding participants with the highest levels of pain-related avoidance.

Prior research has shown psychological inflexibility to be associated with pain-related dysfunction and distress across different types of pain conditions, and the present study provides preliminary evidence that psychological inflexibility also plays a part in the process of adjustment to vulvovaginal pain over time. However, psychological inflexibility was not significantly associated with sexual functioning at baseline, and this finding points to a need to further explore the role of psychological inflexibility in vulvovaginal pain. Previous research has recognized that avoidance behavior may take different forms; it may involve both continued sexual activity and withdrawal from sex. Arguably, the psychological inflexibility model, with its emphasis on the psychological functions of behavior patterns rather than their form, provides a framework that could successfully integrate previous findings relating sexual activity that is motivated by positive outcomes to sexual satisfaction and sexual activity motivated by avoidance of aversive outcomes to reduced psychosexual functioning. In sum, both the findings from the present study and from previous research point to the relevance of further examinations of the psychological inflexibility model in the context of vulvovaginal pain. Future studies should examine the core processes of psychological flexibility and their relationship with sexual well-being, as they may connect women with vulvovaginal pain with positive aspects of sexuality and valued qualities of intimate relationships.

## Notes

Funding sources: None.
